# Epigenetically altered macrophages promote development of diabetes-associated atherosclerosis

**DOI:** 10.3389/fimmu.2023.1196704

**Published:** 2023-05-05

**Authors:** Dong Huang, Wei Gao, Xin Zhong, Hongxian Wu, You Zhou, Yuanji Ma, Juying Qian, Junbo Ge

**Affiliations:** Department of Cardiology, Zhongshan Hospital, Fudan University, Shanghai Institute of Cardiovascular Diseases, Shanghai, China

**Keywords:** atherosclerosis (AS), diabetes, macrophages, endothelial cells, HDAC3, epigenetics

## Abstract

**Background:**

Atherosclerosis (AS) risk is elevated in diabetic patients, but the underlying mechanism such as involvement of epigenetic control of foam macrophages remains unclear. We have previously shown the importance of immune regulation on endothelial cells to AS development in diabetes. In this study, we examined the hypothesis that diabetes may promote AS through modification of the epigenetic status of macrophages.

**Methods:**

We employed the Laser Capture Microdissection (LCM) method to evaluate the expression levels of key epigenetic regulators in both endothelial cells and macrophages at the AS lesions of patients. We then assessed the correlation between the significantly altered epigenetic regulator and serum levels of low-density Lipoprotein (LDL), triglycerides (TRIG) and high-density Lipoprotein (HDL) in patients. *In vitro*, the effects of high glucose on glucose utilization, lactate production, succinate levels, oxygen consumption and polarization in either undifferentiated or differentiated bone marrow-derived macrophages (BMDMs) were analyzed. The effects of depleting this significantly altered epigenetic regulator in macrophages on AS development were assessed in AS-prone diabetic mice.

**Results:**

Histone deacetylase 3 (HDAC3) was identified as the most significantly altered epigenetic regulator in macrophages from the AS lesions in human diabetic patients. The levels of HDAC3 positively correlated with high serum LDL and TRIG, as well as low serum HDL. High glucose significantly increased glucose utilization, lactate production, succinate levels and oxygen consumption in cultured macrophages, and induced proinflammatory M1-like polarization. Macrophage depletion of HDAC3 significantly attenuated AS severity in AS-prone diabetic mice.

**Conclusion:**

Epigenetically altered macrophages promote development of diabetes-associated AS, which could be prevented through HDAC3 depletion.

## Introduction

Diabetes and atherosclerosis (AS) are two closely related conditions that significantly impact global health (1). AS, characterized by the progressive accumulation of inflammatory cells, lipids, and fibrous elements in the arterial wall, is a leading cause of cardiovascular disease (CVD) (2). CVD remains the primary cause of morbidity and mortality in diabetic patients (3). Apolipoprotein E (ApoE) is a potent suppressor of AS. ApoE-knockout (ApoE-KO) mice have been commonly used in research of AS, due to their development of AS features such as hypercholesterolemia in response to a high-fat diet (HFD) (4).

Recent research has emphasized the role of diabetes as a significant risk factor for the development and progression of atherosclerotic lesions (5). The complex crosstalk between foam macrophages and endothelial cells is known to play a crucial role in plaque formation, a hallmark of AS (6–8). The effects of diabetes on AS primarily stem from chronic hyperglycemia, which creates a sustained inflammatory microenvironment that promotes AS development (9). In this microenvironment, macrophages play a vital role, as they not only contribute to plaque formation as foam cells but also actively interact with other cells, such as endothelial cells, mesenchymal cells, fibroblasts, and other immune cells (10). During these interactions, macrophages can change their phenotype or polarization in response to hyperglycemia (11).

Epigenetic changes occur in macrophages and other cell types in diabetes (12). These changes refer to modifications in gene expression without altering the underlying DNA sequence (12), such as histone methyltransferases (HMTs), histone deacetylases (HDACs) and DNA methyltransferases (DNMTs) (13). These enzymes modulate the expression of pro-inflammatory genes, lipid metabolism genes, and cellular adhesion molecules, contributing to the development and progression of atherosclerotic lesions (13). Understanding the specific roles of these epigenetic regulators in diabetes-associated atherosclerosis could help identify novel therapeutic targets for prevention and treatment. Additionally, the interplay between different epigenetic regulators and other factors, such as oxidative stress and advanced glycation end products (AGEs), in the context of diabetes-associated atherosclerosis has been investigated (14).

During the development of AS in diabetes, macrophages undergo epigenetic alterations such as DNA methylation and histone modifications that influence their activation, polarization, and function (12). For example, the promoter region of specific pro-inflammatory genes may exhibit reduced DNA methylation, leading to enhanced expression and contributing to a pro-atherogenic environment (12). Additionally, different post-translational modification may occur to histone proteins that alter chromatin structure and accessibility to affect gene expression (12). In the context of diabetes-associated AS, macrophages may exhibit altered histone modification patterns on genes involved in inflammation, lipid metabolism, and cellular adhesion (15). Increased histone acetylation or methylation on pro-inflammatory genes may promote the expression of these genes, further exacerbating AS (16).

Epigenetic alterations in macrophages have been studied in AS, but not yet in a diabetic setting (12). In this study, we addressed this question by assessing patients’ specimens and examining animal models. Through these approaches, we aimed to uncover the unique epigenetic changes in macrophages that contribute to diabetes-associated AS, providing valuable insights for future research and potential therapeutic interventions.

## Materials and methods

### Study ethics

The study protocol received approval from the Animal Care and Use Committee at Zhongshan Hospital of Fudan University. All experimental procedures followed the guidelines for the Care and Use of Laboratory Animals. Human aortic arch specimens were collected from organ donors or patients who underwent cardiovascular surgery at our institute between 2014 and 2019, with prior consent obtained from the deceased person’s family.

### Animal models

Mice expressing Cre recombinase under the control of the lysosome promoter (pLys-Cre; #004781), histone deacetylase 3 (HDAC3) floxed mice (HDAC3floxed; #024119), and ApoE-KO mice (#002052) were all acquired from the Jackson Laboratory (Bar Harbor, ME, USA) and housed under sterile conditions. In pLys-Cre mice, a nuclear-localized Cre recombinase was inserted at the first coding ATG of the lysozyme 2 gene, thereby abolishing endogenous lysozyme 2 gene function and allowing Cre expression to be controlled by the endogenous lysozyme 2 promoter/enhancer elements (17). In HDAC3floxed mice, loxP sites were positioned flanking exons 4-7 of the HDAC3 gene. Mice homozygous for this allele are viable and fertile. When bred with mice expressing tissue-specific Cre recombinase, the resulting offspring exhibit deletion of exons 4-7 in the Cre-expressing cells (18). The pLys-Cre and HDAC3floxed mice were first crossbred to generate macrophage-specific HDAC3 knockout mice, and then crossbred with ApoE-KO mice to produce ApoE-KO/pLys-Cre/HDAC3floxed mice. ApoE-KO mice (ApoE-KO) (19) were used as controls without macrophage depletion of HDAC3 in ApoE-KO/pLys-Cre/HDAC3floxed mice. Both female and male mice were randomly and equally assigned to each experimental group. AS was induced in ApoE-KO mice by feeding them at age of 8 weeks a high-fat diet (HFD) for 12 weeks to accelerate plaque formation in the arterial wall. The recipe for the HFD contained 21% butter-derived fat by weight and 0.15% cholesterol along with standard rodent chow ingredients. In contrast, control mice are fed a normal diet (ND) that contains 4.5% plant-based fat by weight along with standard rodent chow ingredients. Mice at 20 weeks of age were analyzed for AS development. Four groups of mice were included in the study: ApoE-KO mice with ND, ApoE-KO mice with HFD, ApoE-KO/pLys-Cre/HDAC3floxed mice with ND and ApoE-KO/pLys-Cre/HDAC3floxed mice with HFD.

### Laser capture microdissection

LCM was used to isolate CD31+ endothelial cells and CD68+ macrophages from human aortic arch specimens. Briefly, after collection and processing of the aortic arch tissue, which involved fixation, embedding, and sectioning to generate thin tissue slices, the sections were then mounted on the specialized glass slides for LCM and stained with either rabbit anti-CD31 antibody (Ab28364, Abcam, Shanghai, China) for endothelial cells or rabbit anti-CD68 antibody (Ab213363, Abcam) for macrophages, to visualize and identify the target cell populations. Fibronectin staining (Ab2413, Abcam) was performed to help visualization of the tissue. Before performing LCM, the stained tissue sections were dehydrated through a series of alcohol and xylene washes to remove any residual water and to ensure compatibility with the LCM process. The tissue sections were then placed under the LCM microscope, where the regions containing CD31+ endothelial cells or CD68+ macrophages were carefully identified based on fluorescence. Using an ultraviolet laser, the selected regions are microdissected and captured onto a thermoplastic cap to be transferred to a collection tube for protein extraction and ELISA analysis.

### Quantification of mouse atherosclerotic lesions

The mouse aortic arch dissection and subsequent histological analysis involved several executed steps. First, the aortic arch was carefully removed, ensuring that the tissue remained intact during the process. Following dissection, the aortic arch was fixed in a 4% paraformaldehyde solution for 4 hours to preserve the tissue structure and morphology. After fixation, the tissue was dehydrated through a series of graded alcohol solutions and then embedded in paraffin wax to provide support and protection. Once the tissue was thoroughly infiltrated with paraffin, it was positioned in a mold and allowed to solidify. The paraffin-embedded aortic arch was then sectioned at a thickness of 5 micrometers using a microtome, and the resulting tissue sections were mounted on glass slides for further processing and analysis. To evaluate the presence and extent of atherosclerotic lesions, a Hematoxylin and eosin (H&E) staining was employed. The stained sections were examined under a light microscope. The atherosclerotic lesions were visible as areas of cellular accumulation and extracellular matrix deposition within the intimal layer of the artery wall. The high-resolution images of the stained sections were captured using a microscope equipped with a camera attachment. The atherosclerotic lesion area in each section was outlined with ImageJ analysis software, and the total lesion area was calculated. The lesion area between different treatment groups or experimental conditions was compared to assess the extent of AS.

### Measurement of lipid content of the plaque

To measure the lipid content of an atherosclerotic plaque, we used an Oil Red O staining. First, the aortic arch segments containing atherosclerotic plaques were dissected and then fixed in 4% paraformaldehyde. The fixed tissue was embedded and sectioned into slices of 5μm-thickness, using a cryostat. The sections were fixed in cold acetone for 10 minutes. After that, the slides were stained with freshly prepared Oil Red O solution for 15 minutes. The stained slides were then rinsed with distilled water and counterstained with hematoxylin to visualize nuclei. This staining method selectively stained neutral lipids, such as triglycerides and cholesterol esters, enabling the visualization and quantification of lipid-rich areas within the aortic arch tissue sections. Upon completion of the staining procedures, the slides were examined under a light microscope to assess the presence, distribution, and severity of atherosclerotic lesions in the mouse aortic arch samples.

### Isolation, culture and differentiation of mouse BMDMs

To isolate, culture, and differentiate mouse bone marrow-derived macrophages (BMDMs) into M1 or M2 macrophages, we followed the protocol outlined below. Begin by euthanizing the mice according to institutional guidelines. Spray the hind limbs with 70% ethanol and dissect the femurs and tibias. Remove any attached muscle tissue and transfer the bones to a sterile Petri dish containing phosphate-buffered saline (PBS, Sigma-Aldrich). Cut the ends of the bones using sterile scissors, and flush the bone marrow out with a syringe filled with cold PBS supplemented with 2% fetal bovine serum (FBS). Collect the flushed bone marrow cells in a sterile 50 mL tube. Gently pipette the bone marrow suspension up and down to break up any clumps. Pass the cell suspension through a 70μm cell strainer to remove any debris and obtain a single-cell suspension. Centrifuge the cell suspension at 300 x g for 5 minutes and discard the supernatant. Resuspend the cell pellet in Dulbecco’s Modified Eagle Medium (DMEM, Sigma-Aldrich, Shanghai, China) supplemented with 10% FBS, 1% penicillin-streptomycin, and 25 ng/mL macrophage colony-stimulating factor (M-CSF, Sigma-Aldrich). Count the cells using a hemocytometer and seed them at a density of 10^6^ cells per 10 cm dish or appropriate cell culture plate. Incubate the cells in a humidified 37°C, 5% CO_2_ incubator for 6 days to allow the differentiation of bone marrow cells into macrophages. Replace the culture medium containing M-CSF every 3 days. After the differentiation period, harvest the macrophages using a cell scraper and replate them in new cell culture plates at the desired density. To polarize the macrophages into M1 or M2 phenotypes, treat them with specific stimuli. For M1 polarization, add 100 ng/mL lipopolysaccharide (LPS, Sigma-Aldrich) and 20 ng/mL interferon-gamma (IFN-γ, Sigma-Aldrich) to the culture medium and incubate the cells for 48 hours. For M2 polarization, add 20 ng/mL interleukin-4 (IL-4, Sigma-Aldrich) to the culture medium and incubate the cells for 48 hours.

### Macrophage metabolism

The measurement of macrophage metabolism, including glucose utilization, lactate production, succinate levels, and oxygen consumption rate, was done as following. The undifferentiated or differentiated macrophages were treated with or without experimental stimuli for a specific duration, after which the conditioned media were collected. For glucose utilization, glucose levels were measured with a glucose assay kit according to the manufacturer’s instructions (Ab102517, Abcam). The glucose levels in the media before and after the incubation period were compared to determine glucose utilization by the macrophages. For lactate production, the conditioned media were used to measure lactate levels with a lactate assay kit following the manufacturer’s protocol (Ab65331, Abcam). The amount of lactate produced by the macrophages was used as an indicator of glycolytic activity. For succinate levels, macrophage cell pellets were collected and extracted metabolites using cold methanol as an extraction buffer. The samples were then centrifuged, and the supernatants were collected for quantification of succinate levels using a succinate assay kit, following the manufacturer’s instructions (Ab204718, Abcam). For measuring oxygen consumption rate (OCR) to evaluate the mitochondrial function and oxidative phosphorylation in macrophages, a Seahorse XF Analyzer was used (Aligent, Beijing, China). Seed macrophages in an appropriate cell culture microplate and equilibrate them in a CO_2_-free incubator for 1 hour before analysis, following the manufacturer’s protocol.

### ELISA

RIPA buffer was used to lyse cells to collect proteins. ELISA for human HDAC3 (LS-F55924, LSBio, Seattle, WA, USA), human enhancer of zeste homolog 2 (EZH2; LS-F7278, LSBio), human DNA Methyltransferase 1 (DNMT1; LS-F7340, LSBio), human sirtuin 1 (SIRT1; LS-F12606, LSBio), human jumonji domain containing 3 (JMJD3; LS-F74347, LSBio), mouse IL-6 (Ab100713, Abcam), mouse IL-1β (Ab197742, Abcam), mouse tumor necrosis factor alpha (TNFα; Ab208348, Abcam), mouse interferon gamma (IFNγ; Ab282874, Abcam), mouse arginase 1 (ARG1; Ab269541, Abcam), mouse CD163 (Ab272204, Abcam), mouse α-smooth muscle actin (α-SMA; NBP2-66429, Novus Biologicals, Shanghai, China), Vimentin (LS-F7624, LSBio, Seattle, WA, USA) and mouse Collagen IV (LS-F20750, LSBio) were utilized as per the manufacturer’s instructions.

### Statistical analyses

In this study, the data were presented as individual values along with the mean and standard deviation (SD). The statistical analysis was performed using one-way ANOVA, followed by a Bonferroni *post-hoc* correction to adjust for multiple comparisons (GraphPad Prism, GraphPad Software, Inc., La Jolla, CA, USA). A p-value less than 0.05 was considered statistically significant. The term “NS” was used to denote instances where there was no significant difference between the groups. Additionally, correlation analyses were conducted using Pearson’s correlation coefficient to assess the strength and direction of the relationships between the variables under investigation.

## Result

### Plaque macrophages in diabetic patients express significantly high HDAC3

**Figure 1:**
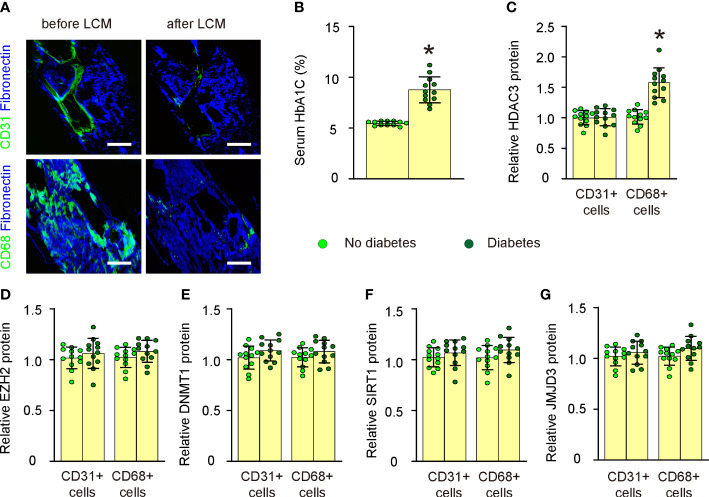
Plaque macrophages in diabetic patients express significantly high HDAC3. **(A)** Representative images for LCM technique to extract either CD31+ endothelial cells or CD68+ macrophages within atherosclerotic lesions of human patients. **(B)** Serum HbA1C percentages in non-diabetic and diabetic groups. **(C–G)** ELISA for HDAC3 **(C)**, EZH2 **(D)**, DNMT1 **(E)**, SIRT1 **(F)**, and JMJD3 **(G)** in CD31+ endothelial cells and CD68+ macrophages from the plaques of both non-diabetic and diabetic cases. *p<0.05. Scale bars were 50µm.

In this study, we utilized the LCM technique to assess the expression levels of key epigenetic regulators in both CD31+ endothelial cells and CD68+ macrophages within atherosclerotic lesions of human patients (**Figure 1A**; **Supplementary Table**). Patient specimens were categorized into non-diabetic and diabetic groups based on their serum HbA1C percentages (**Figure 1B**). We evaluated the expression of five major epigenetic regulators, namely HDAC3, EZH2, DNMT1, SIRT1, and JMJD3, in CD31+ endothelial cells and CD68+ macrophages from the plaques of both non-diabetic and diabetic cases using ELISA (**Figures 1C–G**). While no significant differences were observed in the expression levels of all five epigenetic regulators in CD31+ endothelial cells or in the expression of four of these regulators (EZH2, DNMT1, SIRT1, and JMJD3) in CD68+ macrophages between non-diabetic and diabetic cases (**Figures 1C–G**), we detected a notable increase in HDAC3 levels in macrophages from diabetic patients compared to those from non-diabetic individuals (**Figure 1C**). Thus, plaque macrophages in diabetic patients exhibit significantly elevated HDAC3 expression.

### Macrophage HDAC3 levels correlate with lipid metabolism and glucose levels in patients

**Figure 2:**
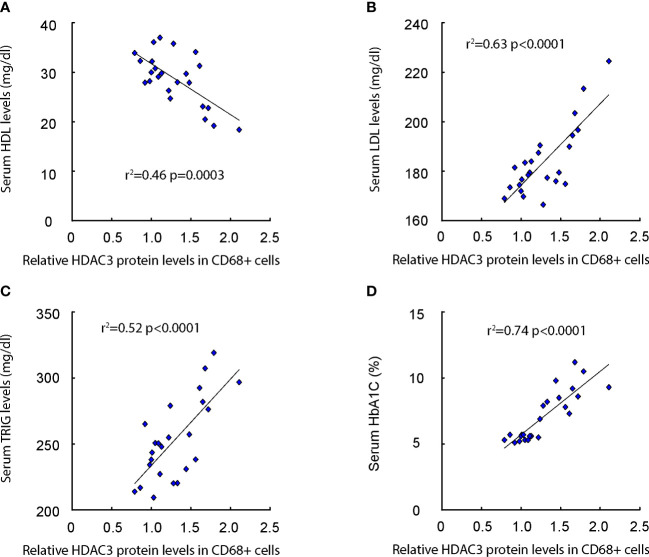
Macrophage HDAC3 levels correlate with lipid metabolism and glucose levels in patients. **(A–D)** The correlation between macrophage HDAC3 expression levels and serum Low-density Lipoprotein (LDL; **A**), High-density Lipoprotein (HDL; **B**), Triglycerides (TRIG; **C**) and HbA1C% **(D)** in patients.

We subsequently evaluated the correlation between macrophage HDAC3 expression levels and lipid metabolic parameters, such as serum Low-density Lipoprotein (LDL), High-density Lipoprotein (HDL), Triglycerides (TRIG), as well as glucose levels (HbA1C%) in patients (**Supplementary Table**). Our findings revealed a negative correlation between macrophage HDAC3 levels and serum HDL (**Figure 2A**), while a positive correlation was observed with serum LDL (**Figure 2B**) and TRIG (**Figure 2C**). Additionally, a positive correlation was found between macrophage HDAC3 levels and patients’ blood glucose levels (**Figure 2D**). These results, demonstrating a correlation between macrophage HDAC3 expression and lipid metabolism as well as glucose levels in patients, suggest that HDAC3-mediated epigenetic alterations in macrophages may contribute to the metabolic changes associated with AS in diabetes.

### High glucose increases macrophage metabolism

**Figure 3:**
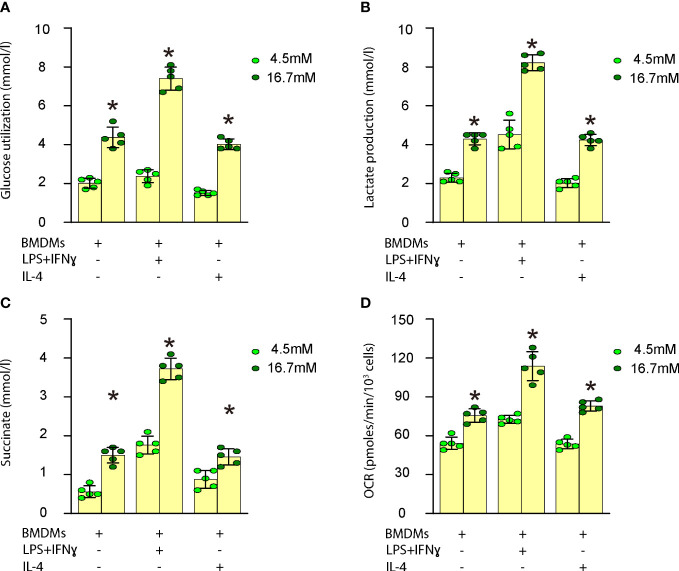
High glucose increases macrophage metabolism. Measurement of the impact of high glucose on glucose utilization, lactate production, succinate levels and oxygen consumption in both undifferentiated and differentiated mouse BMDMs. **(A)** Glucose utilization assay **(B)** Lactate production assay **(C)** Succinate levels **(D)** Oxygen consumption rate (OCR) in undifferentiated BMDMs as well as in differentiated BMDMs (M1 by LPS and IFNγ; M2 by IL-4). *p<0.05.

*In vitro*, we investigated the impact of high glucose on glucose utilization, lactate production, succinate levels, oxygen consumption, and polarization in both undifferentiated and differentiated mouse BMDMs. Our findings demonstrated that high glucose significantly enhanced glucose utilization (**Figure 3A**), lactate production (**Figure 3B**), succinate levels (**Figure 3C**), and oxygen consumption (**Figure 3D**) in undifferentiated BMDMs as well as in differentiated BMDMs (M1 by LPS and IFNγ; M2 by IL-4). These results indicate that elevated glucose levels boost macrophage metabolism, which may contribute to their activation and involvement in plaque formation.

### High glucose induces proinflammatory polarization of macrophages

**Figure 4:**
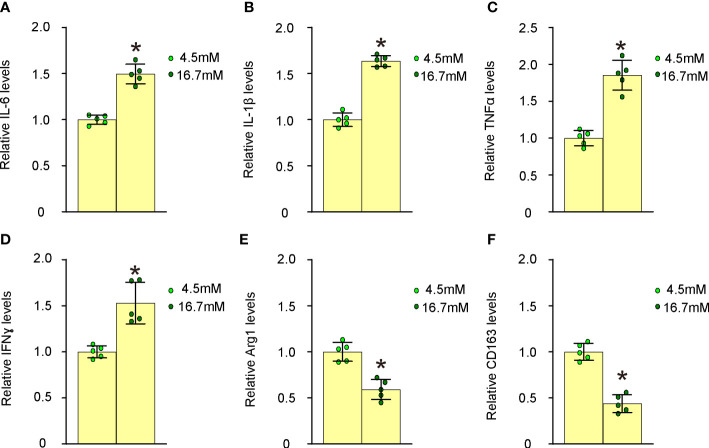
High glucose induces proinflammatory polarization of macrophages. The effects of high glucose on mouse macrophage polarization were assessed by evaluating the expression levels of several critical M1/M2 macrophage markers using ELISA. **(A)** ELISA for IL-6 **(B)** ELISA for IL-1β **(C)** ELISA for TNFα **(D)** ELISA for IFNγ **(E)** ELISA for Arg1 **(F)** ELISA for CD163. *p<0.05.

We proceeded to examine the effects of high glucose on macrophage polarization by evaluating the expression levels of several critical M1/M2 macrophage markers. Our findings revealed that high glucose significantly upregulated M1 markers, such as IL-6 (**Figure 4A**), IL-1β (**Figure 4B**), TNFα (**Figure 4C**), and IFNγ (**Figure 4D**), while it notably downregulated M2 markers, including Arg1 (**Figure 4E**) and CD163 (**Figure 4F**), in undifferentiated BMDMs. These observations suggest that elevated glucose levels promote proinflammatory polarization of macrophages, which may contribute to their transformation into foam cells and subsequent plaque formation.

### Generation of macrophage HDAC3 knockout mice in ApoE-KO background

**Figure 5:**
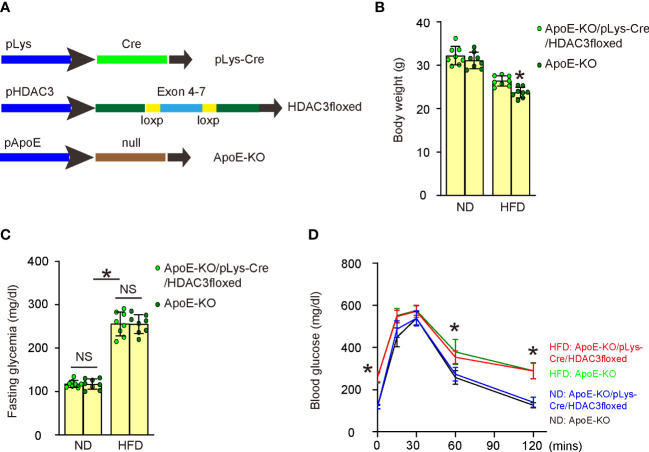
Generation of macrophage HDAC3 knockout mice in ApoE-KO background. **(A)** Illustration of generating macrophage HDAC3 knockout mice in an ApoE-KO background. In pLys-Cre mice, the promoter of the macrophage-specific endogenous lysozyme 2 gene was utilized to drive Cre recombinase expression. In HDAC3floxed mice, loxP sites were placed flanking exons 4-7 of the HDAC3 gene to facilitate Cre-mediated HDAC3 depletion. We first crossbred pLys-Cre and HDAC3floxed mice to generate macrophage-specific HDAC3 knockout mice and then crossed them with ApoE-KO mice to produce ApoE-KO/pLys-Cre/HDAC3floxed mice. ApoE-KO mice were used as controls without macrophage depletion of HDAC3 in ApoE-KO/pLys-Cre/HDAC3floxed mice. Both strains of mice were fed either a high-fat diet (HFD) or a normal diet (ND), resulting in four experimental groups: ApoE-KO mice with ND, ApoE-KO mice with HFD, ApoE-KO/pLys-Cre/HDAC3floxed mice with ND, and ApoE-KO/pLys-Cre/HDAC3floxed mice with HFD. **(B–D)** Body weights **(B)**, fasting blood sugar **(C)** and IPGTT **(D)** after 12 weeks of ND/HFD treatment. *p<0.05. NS: no significance.

To investigate whether increased HDAC3 expression in macrophages might contribute to the development of AS *in vivo*, we generated macrophage HDAC3 knockout mice in an ApoE-KO background. In pLys-Cre mice, the promoter of the macrophage-specific endogenous lysozyme 2 gene was utilized to drive Cre recombinase expression (17). In HDAC3floxed mice, loxP sites were placed flanking exons 4-7 of the HDAC3 gene to facilitate Cre-mediated HDAC3 depletion (18). We first crossbred pLys-Cre and HDAC3floxed mice to generate macrophage-specific HDAC3 knockout mice and then crossed them with ApoE-KO mice to produce ApoE-KO/pLys-Cre/HDAC3floxed mice. ApoE-KO mice were used as controls without macrophage depletion of HDAC3 in ApoE-KO/pLys-Cre/HDAC3floxed mice. Both strains of mice were fed either a high-fat diet (HFD) or a normal diet (ND), resulting in four experimental groups: ApoE-KO mice with ND, ApoE-KO mice with HFD, ApoE-KO/pLys-Cre/HDAC3floxed mice with ND, and ApoE-KO/pLys-Cre/HDAC3floxed mice with HFD (**Figure 5A**). After 12 weeks of ND/HFD treatment, we observed that ND did not lead to changes in body weights between ApoE-KO and ApoE-KO/pLys-Cre/HDAC3floxed mice, while HFD resulted in significantly higher body weights in ApoE-KO/pLys-Cre/HDAC3floxed mice compared to ApoE-KO mice (**Figure 5B**). Furthermore, no differences were found in fasting blood sugar (**Figure 5C**) or glucose responses (**Figure 5D**) between the two strains when treated with either ND or HFD. Subsequently, these mice were analyzed for the development and severity of AS.

### Macrophagic depletion of HDAC3 attenuates severity of AS in HFD-treated ApoE-KO mice

**Figure 6:**
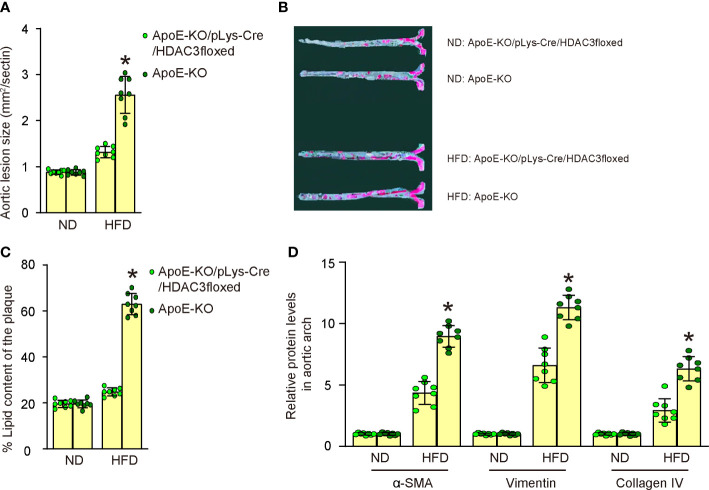
Macrophagic depletion of HDAC3 attenuates severity of AS in HFD-treated ApoE-KO mice. **(A)** Quantification of aortic lesion size in the aortic sinus of HFD-treated mice compared to ND-treated mice in both strains. **(B, C)** Oil Red O staining to assess the plaque lipid content in HFD-treated mice compared to ND-treated mice in both strains, shown by representative images **(B)** and by quantification **(C)**. **(D)** ELISA for mesenchymal markers α-SMA, Vimentin, and Collagen IV of plaque tissue. *p<0.05.

We assessed the impact of HDAC3 depletion in macrophages on the development of AS in these diabetes-prone, AS-susceptible mice. We observed a significant increase in aortic lesion size in the aortic sinus of HFD-treated mice compared to ND-treated mice in both strains (**Figure 6A**). However, macrophage-specific HDAC3 depletion significantly reduced the aortic lesion size in HFD-treated mice (**Figure 6A**). Additionally, the aortic sinus exhibited a significant increase in plaque lipid content in HFD-treated mice compared to ND-treated mice in both strains (**Figures 6B, C**). Nevertheless, macrophage-specific HDAC3 depletion substantially reduced plaque lipid content in HFD-treated mice (**Figures 6B, C**). We then isolated the aortic arch to measure the levels of mesenchymal markers α-SMA, Vimentin, and Collagen IV using ELISA. We detected significantly elevated levels of α-SMA, Vimentin, and Collagen IV in HFD-treated mice in both strains. However, these increases in all mesenchymal markers were significantly attenuated in HFD-treated ApoE-KO/pLys-Cre/HDAC3floxed mice compared to ApoE-KO mice (**Figure 6D**). Collectively, these findings suggest that macrophage-specific HDAC3 depletion significantly ameliorates AS severity in diabetes-prone, AS-susceptible mice.

## Discussion

Epigenetic alterations in macrophages during the development of AS in diabetes contribute to the overall disease pathogenesis by modulating macrophage function, promoting inflammation, and facilitating plaque formation and progression (12). Understanding more deeply of these specific epigenetic changes and their functional consequences may offer novel therapeutic targets for preventing and treating diabetes-associated AS (12).

In fact, the crosstalk between foam macrophages and endothelial cells constitutes a central component of plaque formation (20). Hyperglycemia, a primary characteristic of diabetes, contributes to endothelial dysfunction, which is considered an early and crucial event in AS pathogenesis (21–25). Endothelial dysfunction increases endothelial monolayer permeability, enabling the infiltration of LDL particles into the arterial intima (26). Once inside the intima, LDL particles become susceptible to oxidative modifications, generating pro-inflammatory and pro-atherogenic oxidized LDL (oxLDL) (27). Hyperglycemia-induced endothelial dysfunction, along with the pro-inflammatory and pro-atherogenic effects of oxLDL and advanced glycation end products (AGEs), drives the recruitment, activation, and transformation of macrophages into foam cells within the arterial wall (28). Our study showed that the relationship between diabetes and AS may be partially mediated by epigenetic modifications of macrophages. Macrophages respond to high blood glucose levels, altering their epigenetic status and impacting the microenvironment to facilitate AS development.

The interaction between foam macrophages and endothelial cells in the context of diabetes has also been shown to be influenced by AGEs, which form through non-enzymatic reactions between glucose and proteins, lipids, or nucleic acids (29). AGEs can bind to their receptor (RAGE) on both endothelial cells and macrophages, activating intracellular signaling pathways that promote inflammation, oxidative stress, and vascular remodeling, thereby exacerbating AS development (29). In this study, we found that hyperglycemia-induced epigenetic alterations in macrophages were likely mediated by HDAC3, which modifies macrophage metabolism and polarization, contributing to AS. It would be interesting for future research to examine the effects of epigenetic changes in macrophages on AGE expression and functionality and on the molecular pathways downstream of HDAC3 that are involved in macrophage polarization, metabolism, and function in the context of diabetes-associated atherosclerosis, such as NK-kB signaling (30), peroxisome proliferator-activated receptor gamma (PPARγ) signaling (31), STAT signaling (32), or mTor signaling (33). For example, a previous study has shown that sustained high expression of HDAC3 can inhibit the expression of PPARγ, which could contribute to the HDAC3-induced M1 macrophage polarization (31).

Here, the molecular mechanism underlying increased lactate production, succinate levels, and OCR in M1 BMDMs stimulated with LPS and IFN-γ involved shifting of their metabolic profile towards glycolysis and exhibition of augmented activity of the tricarboxylic acid (TCA) cycle (34). Increased lactate production should result from the upregulation of glycolytic enzymes, including hexokinase, phosphofructokinase, and pyruvate kinase, which accelerate glycolysis. The upregulation of lactate dehydrogenase (LDH) promotes the conversion of pyruvate to lactate, leading to increased lactate secretion (34). The accumulation of succinate, an intermediate of the TCA cycle, was a result of the increased activity of enzymes like succinate dehydrogenase (SDH), which converts succinate to fumarate (34). Increased OCR in M1 macrophages reflects the higher metabolic demands of these cells, which exhibit increased mitochondrial respiration, partially fueled by the breakdown of fatty acids through β-oxidation (34). Thus, the molecular mechanisms underlying the metabolic changes in LPS+IFN-γ-induced M1 BMDMs involve the upregulation of glycolysis, an altered TCA cycle with the accumulation of succinate, and increased mitochondrial respiration, which together support the pro-inflammatory function of M1 macrophages.

The depletion of HDAC3 in macrophages can have significant effects on macrophage function, including alterations in macrophage polarization, metabolism, and key metabolic parameters such as lactate production, succinate levels, OCR, and glucose consumption (35). First, HDAC3 plays a crucial role in regulating gene expression through histone deacetylation. Depletion of HDAC3 may lead to changes in the expression of genes involved in macrophage polarization. This could result in a shift in macrophage phenotype, potentially promoting an anti-inflammatory M2 phenotype over a pro-inflammatory M1 phenotype (35). Moreover, HDAC3 regulates metabolic pathways in macrophages, and its depletion could alter the balance between glycolysis and oxidative phosphorylation, the accumulation of TCA cycle intermediates, such as succinate, and the mitochondrial respiration and oxidative phosphorylation (35). All these effects may lead to changes in the macrophage’s metabolic profile and impact its ability to respond to inflammatory stimuli or perform tissue repair functions (35). Thus, the depletion of HDAC3 in macrophages may influence the macrophage’s capacity to mount an appropriate immune response and contribute to the progression or resolution of inflammation and associated pathologies.

Besides high glucose, insulin resistance and dyslipidemia are also key factors in diabetes that significantly impact macrophage function and AS progression (36). Insulin resistance impairs glucose uptake, leading to chronic hyperglycemia, which creates a pro-inflammatory environment that exacerbates macrophage activation and polarization toward the M1 pro-inflammatory phenotype (37). Dyslipidemia, characterized by elevated levels of LDL and decreased levels of HDL, promotes the accumulation of lipids in macrophages, forming foam cells that contribute to plaque development (38). The interaction between insulin resistance and dyslipidemia synergistically promotes inflammation, oxidative stress, and endothelial dysfunction, further driving macrophage activation and AS progression in diabetes (39). Figure studies may investigate the impact of insulin resistance and dyslipidemia in the setting of this study.

While the ApoE-KO mouse model is widely used in AS research, it does not fully recapitulate all pathological changes observed in human AS (40). Since each animal model has its advantages and disadvantages, future work should utilize additional animal models to validate the findings of this study before translating them to clinical research. Moreover, the findings in this study could be strengthened by examining the potential therapeutic effects of pharmacological inhibition or modulation of HDAC3 activity in preclinical models of diabetes-associated atherosclerosis.

In summary, our study identified a specific role for macrophage HDAC3 in regulating AS development through epigenetic modification of macrophages and may offer new insights for the development of innovative AS therapies.

## Data availability statement

The original contributions presented in the study are included in the article/**Supplementary Material**. Further inquiries can be directed to the corresponding authors.

## Ethics statement

The studies involving human participants were reviewed and approved by Zhongshan Hospital, Fudan University. The patients/participants provided their written informed consent to participate in this study. The animal study was reviewed and approved by Zhongshan Hospital, Fudan University.

## Author contributions

DH and JG designed the study and applied for grant supports. DH, WG, XZ, HW, YZ, YM, JQ and JG did data acquisition and analysis. DH wrote the manuscript. All authors reviewed the manuscript and agreed with the publication. JG is the guarantee of this study. All authors contributed to the article and approved the submitted version.
